# Research progress of breast cancer surgery during 2010–2024: a bibliometric analysis

**DOI:** 10.3389/fonc.2024.1508568

**Published:** 2024-12-09

**Authors:** Jiawei Kang, Nan Jiang, Munire Shataer, Tayier Tuersong

**Affiliations:** ^1^ Department of Clinical Medicine, Xinjiang Medical University, Ürümqi, Xinjiang, China; ^2^ Department of Histology and Embryology, Basic Medical College of Xinjiang Medical University, Ürümqi, Xinjiang, China; ^3^ Department of Pharmacy, Xinjiang Key Laboratory of Neurological Diseases, Xinjiang Clinical Research Center for Nervous System Diseases, Second Affiliated Hospital of Xinjiang Medical University, Ürümqi, Xinjiang, China

**Keywords:** breast cancer surgery, bibliometric analysis, research hotspots, international collaboration, publication patterns

## Abstract

**Purpose:**

This study seeks to systematically analyze the research literature pertaining to breast cancer surgery from 2010 to 2024, as indexed in the PubMed database, employing bibliometric methodologies.

**Methods:**

Employing the “bibliometrix” package in the R programming language, alongside VOSviewer and CiteSpace software, this research conducted a comprehensive visual analysis of 1,195 publications. The analysis encompassed publication trends, collaborative networks, journal evaluation, author and institutional assessments, country-specific analyses, keyword exploration, and the identification of research hotspots.

**Results:**

The study observed a rising trend in the number of publications related to breast cancer surgery. However, there was a concomitant decline in citation rates, potentially indicating either a saturation of the research field or a diminution in research quality. The United States, China, and Japan are the leading contributors to research output, with the United States showing the most extensive international collaboration. The University of California, University of Toronto, and University of Texas MD Anderson Cancer Center were the top institutions for the number of published papers. Through a comprehensive analysis of keywords, we have identified “breast cancer” “pain” “anxiety” “lymphedema” “mastectomy” and “surgery” as central research themes within this domain, the corresponding clusters were subjected to analysis.

**Conclusion:**

This study provides a comprehensive review of breast cancer surgery research, emphasizing major research areas and proposing future research directions. This study provides a significant resource for researchers and clinicians in the field.

## 1 Introduction

Breast cancer (BC) is a leading malignancy among women globally and significantly contributes to cancer-related mortality in this group. Surgical intervention, a fundamental aspect of the comprehensive therapeutic approach to BC (1), plays an indispensable role. An examination of surgical trends from 2005 to 2017 indicates a decline in the preference for invasive surgical procedures, accompanied by an increase in breast-conserving surgery (BCS) and reconstructive surgery (2). Increasing evidence indicates that BCS, when combined with adjuvant therapy, not only preserves the efficacy of tumor treatment but also improves aesthetic outcomes and patient satisfaction (3). This advancement can be traced back to the pioneering work of Umberto Veronesi, whose dedicated research and clinical practice significantly enhanced patient treatment experiences and quality of life, while also establishing a robust foundation for future research and clinical applications (4, 5). BC surgery has significantly evolved since the mid-20th century, transitioning from radical mastectomy to more conservative techniques that emphasize patient quality of life. This transition is largely attributed to the pioneering research and clinical trials conducted by Dr. Bernard Fisher (6). Recent investigations have examined novel surgical methodologies, including opioid-free anesthesia, that enhance postoperative recovery while maintaining effective pain management (7).

Bibliometrics, a discipline dedicated to the quantitative analysis of scientific literature, has emerged as an invaluable instrument for uncovering research trends, identifying knowledge gaps within research domains, and evaluating the impact of scientific contributions (8). Through the visualization of research patterns and collaboration networks, bibliometrics offers a distinctive perspective on the dynamics of the scientific community (9). A comprehensive bibliometric analysis of literature pertaining to BC surgery can identify emerging research themes, landmark studies, and the geographical distribution of scientific contributions (10).

This study aims to perform a detailed bibliometric examination of publications on BC surgery from 2010 to 2024.Utilizing the PubMed database, this research seeks to elucidate trends, prominent topics, and their developmental trajectories within this domain. This analysis examines publication trends, collaboration patterns, and the principal contributors to the field, categorized by countries, institutions, journals, and authors. Furthermore, the study explores prominent research topics and developmental trends in BC surgery, with the objective of offering guidance and insights for future research directions and clinical practices.

## 2 Methods

### 2.1 Data sources and retrieval strategy

PubMed, an integral component of the NCBI’s Entrez search system, offers access to 38 distinct databases, thereby serving as a central repository for medical literature. As of December 2023, PubMed contains over 36 million publications, underscoring its extensive collection of medical research (11). Data for this study were sourced from the PubMed database. On August 7, 2024, we exported a dataset of literature, complete with metadata, utilizing the “PubMed export file” feature (12). Our retrieval strategy was based on a topic search:((Breast Cancer Surgery[title]) AND ((“2010/1/1”[Date - Publication]: “2024/8/7”[Date – Publication])).

### 2.2 Data analysis

We conducted a bibliometric analysis of the collected data using the “bibliometrix” package in R (version 4.3.1, http://www.bibliometrix.org) (13). This package enabled the extraction of critical information and the construction of co-occurrence networks encompassing countries, institutions, journals, and authors. Furthermore, it facilitated thematic evolution analysis and the development of a global publication distribution network. In addition, VOSviewer software was employed for the visualization of collaboration and co-word networks (14, 15). CiteSpace (version 5.8.R2) was employed to analyze keyword co-occurrence and citation networks, revealing research trends and knowledge structures (16).

## 3 Results

### 3.1 Literature search and processing

**Figure 1:**
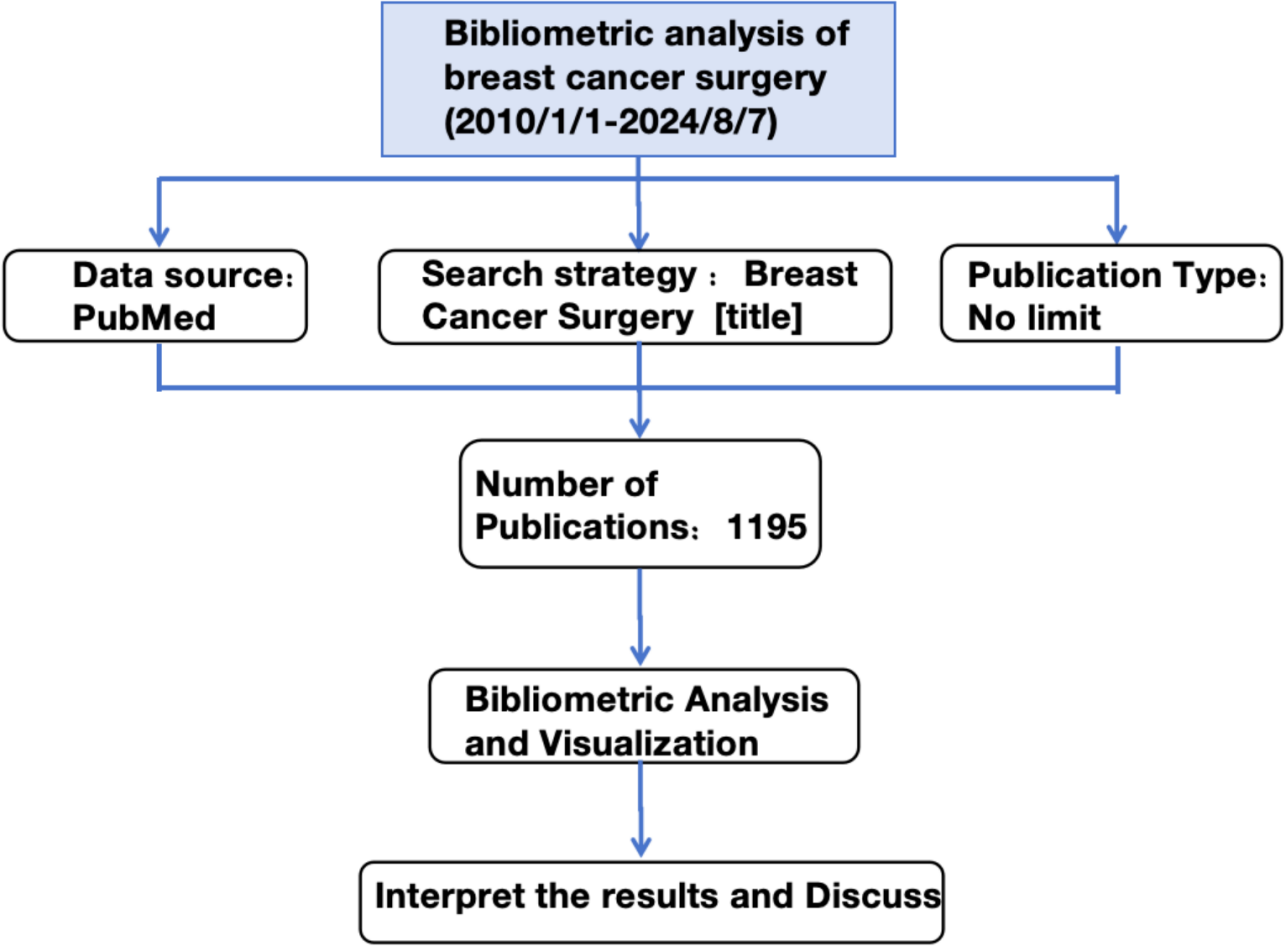
Workflow of the study.

A thorough literature review identified 1,195 articles. **Figure 1** provides a comprehensive overview of the methodology.

### 3.2 Analysis of annual publishing trends and citation volume dynamics

**Figure 2:**
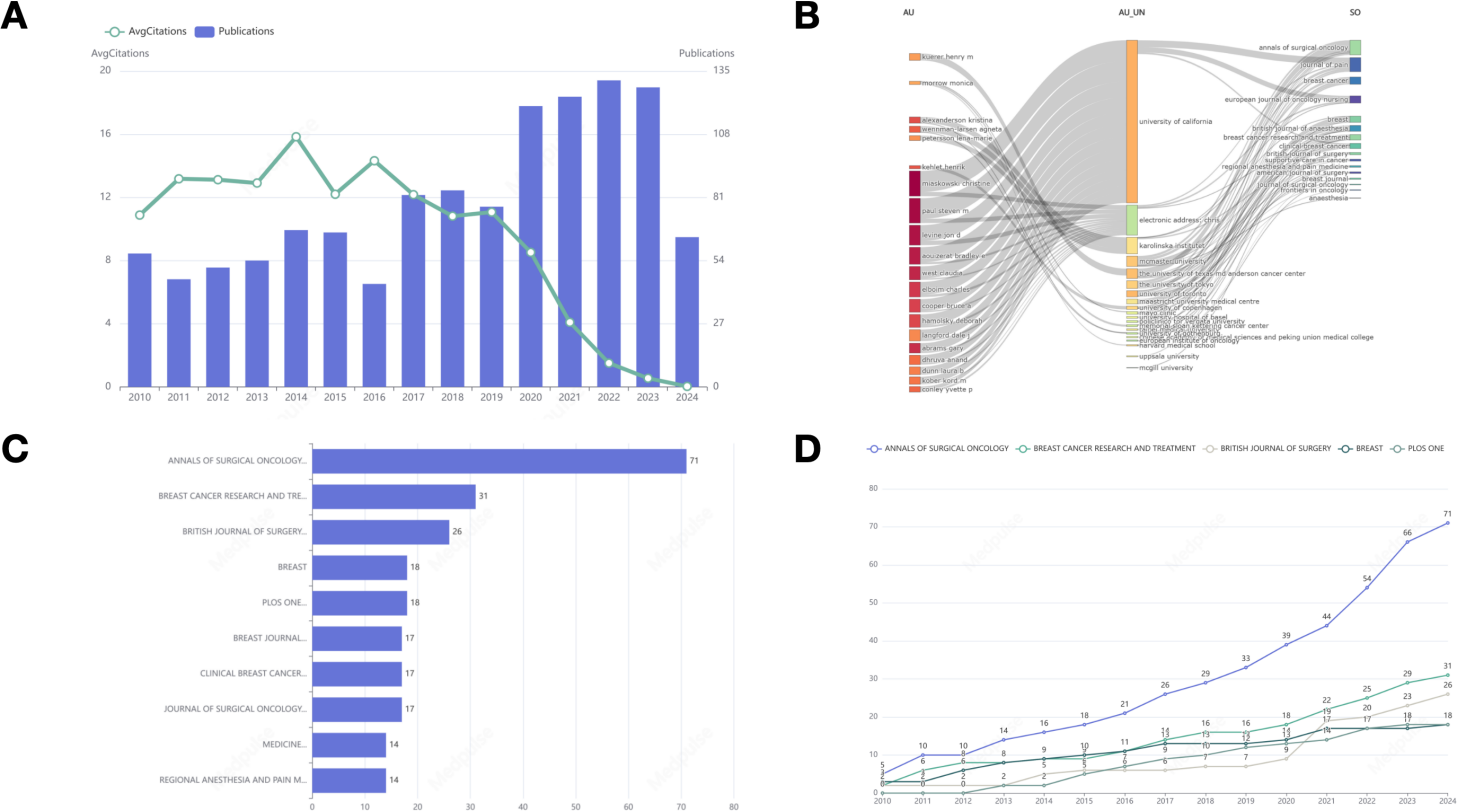
**(A)** Annual publication trends and average annual citations. **(B)** Three-field plot, Left field: Author; Middle field: Affiliation; Right field: Source. **(C)** Bar graph representing the distribution of literature sources. **(D)** The statistics of the top five journals in terms of cumulative publication volume in different years.

**Figure 2A** presents the total number of relevant articles retrieved, which amounts to 1,195. The average annual publication rate is approximately 79 articles per year. In 2010, the scholarly output pertaining to the subject comprised 57 publications. Subsequently, a gradual increase was observed, culminating in a peak of 120 articles by 2020. However, a slight decline occurred in 2021, succeeded by a substantial rise in 2022 and 2023, with publication counts reaching 124 and 131 articles, respectively. Notably, in 2024, there was a pronounced decrease in the number of publications, with only 64 articles released. Furthermore, the trend in average annual citation counts demonstrates an inverse relationship with the trend in publication volume.

### 3.3 Three-field plot

**Figure 2B** illustrates a Sankey diagram that represents the interconnections among authors, their affiliations, and the sources of publication. A comprehensive analysis of the collaborative networks among these entities is undertaken, revealing the University of California as a pivotal institution within these partnerships.

### 3.4 Journal analysis

**Figure 2C** displays a ranking of the top ten journals based on publication volume. The ANNALS OF SURGICAL ONCOLOGY leads with 71 publications, followed by BREAST CANCER RESEARCH AND TREATMENT with 31, and the BRITISH JOURNAL OF SURGERY with 26. **Figure 2D** illustrates the annual publication counts for the top five journals in BC surgery. Recent years have seen an overall increase in publication numbers in prominent journals, with the ANNALS OF SURGICAL ONCOLOGY experiencing notable growth.

### 3.5 Author analysis

**Figure 3:**
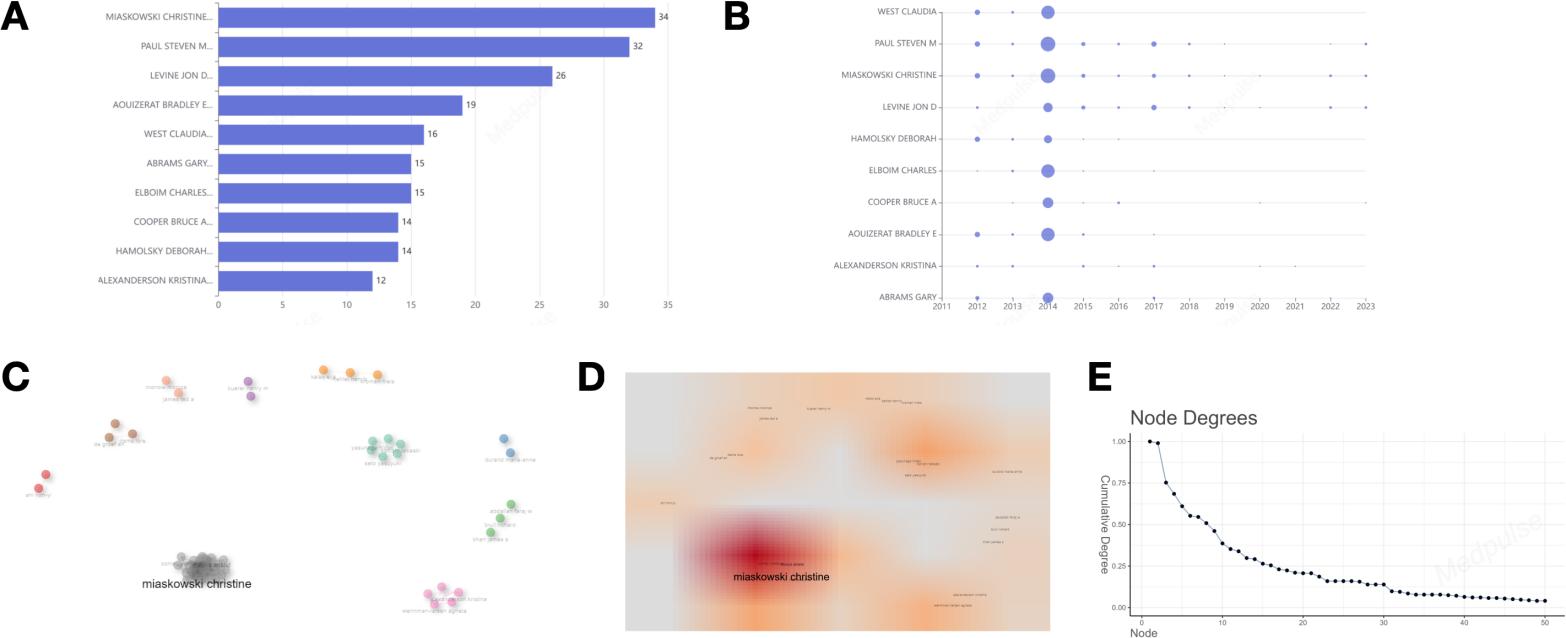
**(A)** Ten of the most relevant authors. **(B)** Top 10 authors’ production over time. **(C–E)** Collaboration network map and density map of the key authors analysis. **(C)** Collaboration network map. **(D)** Density map; **(E)** The number of connections per node in the network.

**Figure 3A** displays a ranking of the top ten authors based on their number of publications. MIASKOWSKI CHRISTINE is the leading author with 34 publications, followed by PAUL STEVEN M with 32 publications, and LEVINE JON D, who holds the third position with 26 publications. **Figure 3B** depicts the annual publication output of these authors, emphasizing the academic productivity of MIASKOWSKI CHRISTINE, PAUL STEVEN M, AOUIZERAT BRADLEY E, WEST CLAUDIA, ABRAMS GARY, HAMOLSKY DEBORAH, and LEVINE JON D in the years 2012 and 2014, as detailed in **Table 1**. Each of these authors demonstrated a remarkable scholarly output, publishing no fewer than 120 works annually over the two-year period. Furthermore, Elboim Charles and Cooper Bruce A also showed notable productivity in 2014. **Figures 3C–E** illustrate the collaborative network among the authors. In this network diagram, the connections between nodes signify collaborative efforts, underscoring that authors with the highest publication counts have increasingly engaged in collaborative endeavors with their peers in recent years. Notably, Miaskowski Christine emerges as the most interconnected author, with a betweenness centrality of 3.9754, a closeness centrality of 0.0556, and a PageRank of 0.0409, this suggests that his extensive collaborative efforts may position him as a pivotal researcher within the field.

**Table 1:** The top 9 authors published the most in 2012 and 2014.

Authors	Number of Publications	
	2012	2014
MIASKOWSKI CHRISTINE	165	262
PAUL STEVEN M	165	262
AOUIZERAT BRADLEY E	165	239
WEST CLAUDIA	165	239
ABRAMS GARY	147	215
HAMOLSKY DEBORAH	165	120
LEVINE JON D	122	135
ELBOIM CHARLES	54	247
COOPER BRUCE A	0	190

### 3.6 Institution analysis

**Figure 4:**
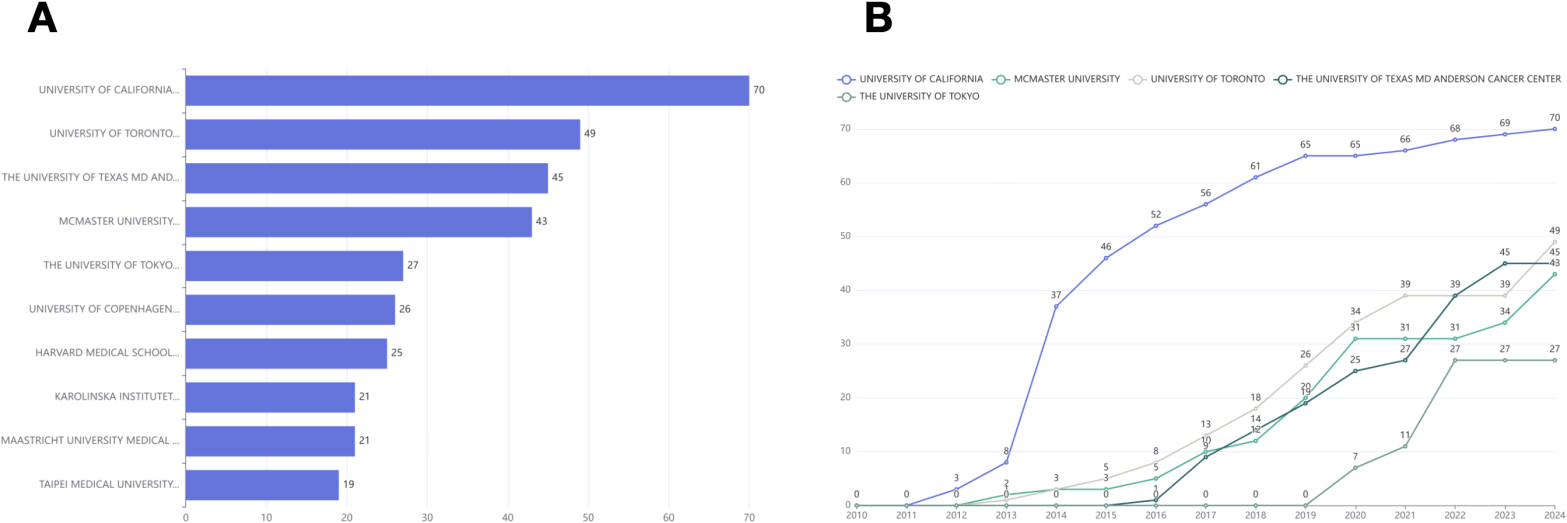
**(A)** Top 10 most relevant affiliations. **(B)** The top 5 affiliations’ production over time.

**Figure 4A** shows that the University of California has the most publications (n=70). The University of Toronto ranks second with 49 publications, followed by The University of Texas MD Anderson Cancer Center in third place with 45 publications. **Figure 4B** depicts the publication trends over the years for the top five institutions, highlighting the University of California as the most consistent and significant contributor. Significant contributions to the field have been made by the University of Toronto, McMaster University, the University of Texas MD Anderson Cancer Center, and the University of Tokyo. An increasing trend in the number of publications is evident across all these institutions.

### 3.7 National analysis

**Figure 5:**
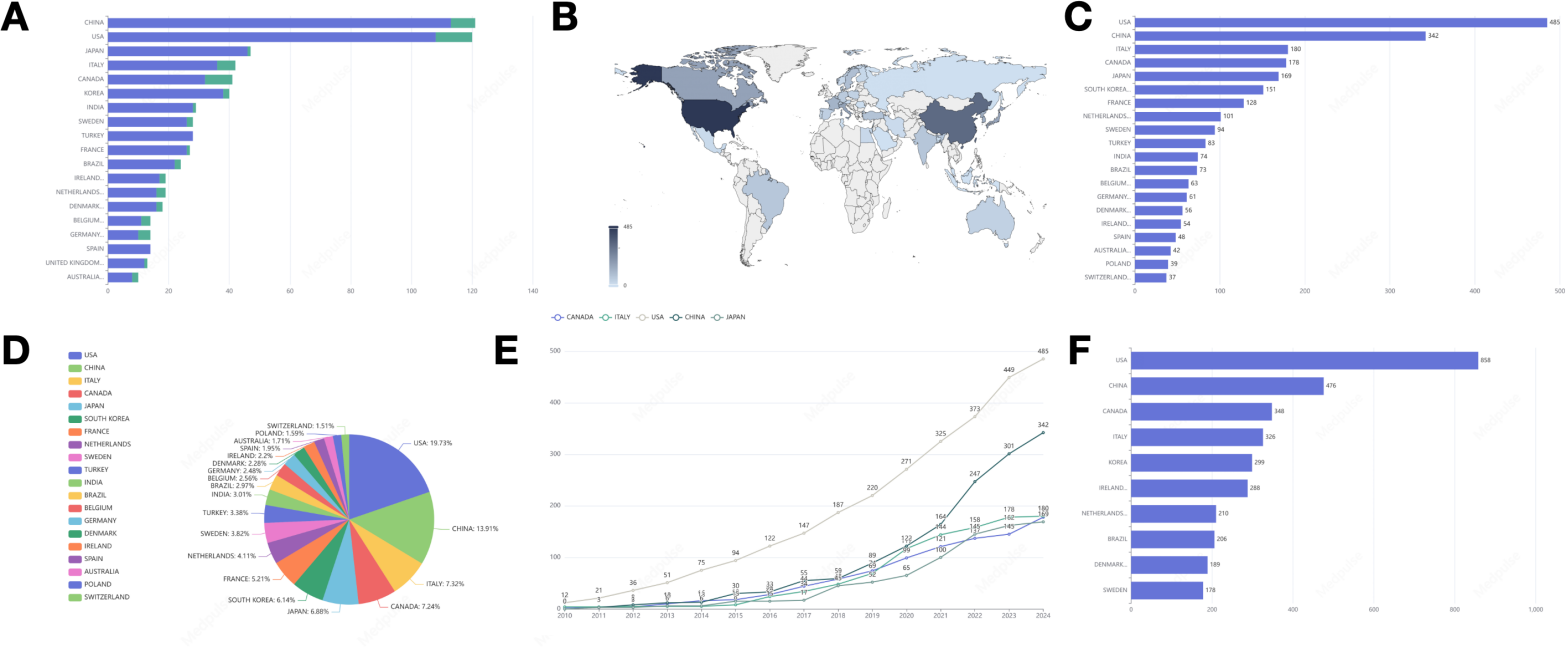
**(A)** Ranking of publications in the countries where the corresponding authors are located and the proportion of international cooperation (blue for single-country publications, green for multi-country publications). **(B)** Country scientific production. **(C)** Distribution of publications in the top 20 countries. **(D)** Percentage distribution of publications in the top 20 countries. **(E)** The number of publications in the top 5 countries by volume and their growth trend. **(F)** The most influential country in terms of number of citations to its articles.

In our analysis of national contributions, corresponding authors originate from 19 different countries. **Figure 5A** presents a ranking of these countries based on their publication output, with China at the forefront (P=121; SCP=113; MCP=8), followed by the United States (P=120; SCP=108; MCP=12), and Japan in third place (P=47; SCP=46; MCP=1). The majority of publications originating from these countries are characterized by multinational collaborations. In contrast, Spain exhibits a multinational publication ratio of 0%, indicating a notable absence of international cooperation. China and the United States demonstrate multinational publication ratios of 6.81% and 10%, respectively, suggesting that China’s level of international collaboration is comparatively lower than that of the United States.

The academic publications analyzed in this study are distributed across 60 countries and regions worldwide, as depicted in **Figure 5B**. We have mapped the geographical distribution of these publications, with a particular focus on the top 20 countries, as illustrated in **Figures 5C, D**. Among these, 12 countries are from Europe, along with several from North America, South America, Asia, and Oceania. The United States ranks first in publications with 485 entries (19.73%), followed by China with 342 entries (13.91%). Italy ranks third in publication output (n=180, p=7.32%).

Upon analyzing the trends in publication growth across various countries, it is evident that the top five nations, in terms of publication volume, exhibit a consistent annual increase, as illustrated in **Figure 5E**. The United States leads in contributions with the most publications, followed by China, Italy, Canada, and Japan. It is noteworthy that China has experienced a marked upward trajectory in publication output since 2021.

**Figure 5F** illustrates the citation impact of the top ten countries by total citations (TC). The top three countries include the United States, China, and Canada. The United States exhibits a notably higher total number of citations (TC=858) compared to other nations, being approximately 1.8 times greater than China (TC=476) and 2.5 times greater than Canada (TC=348).

### 3.8 Countries’ collaboration world map

**Figure 6:**
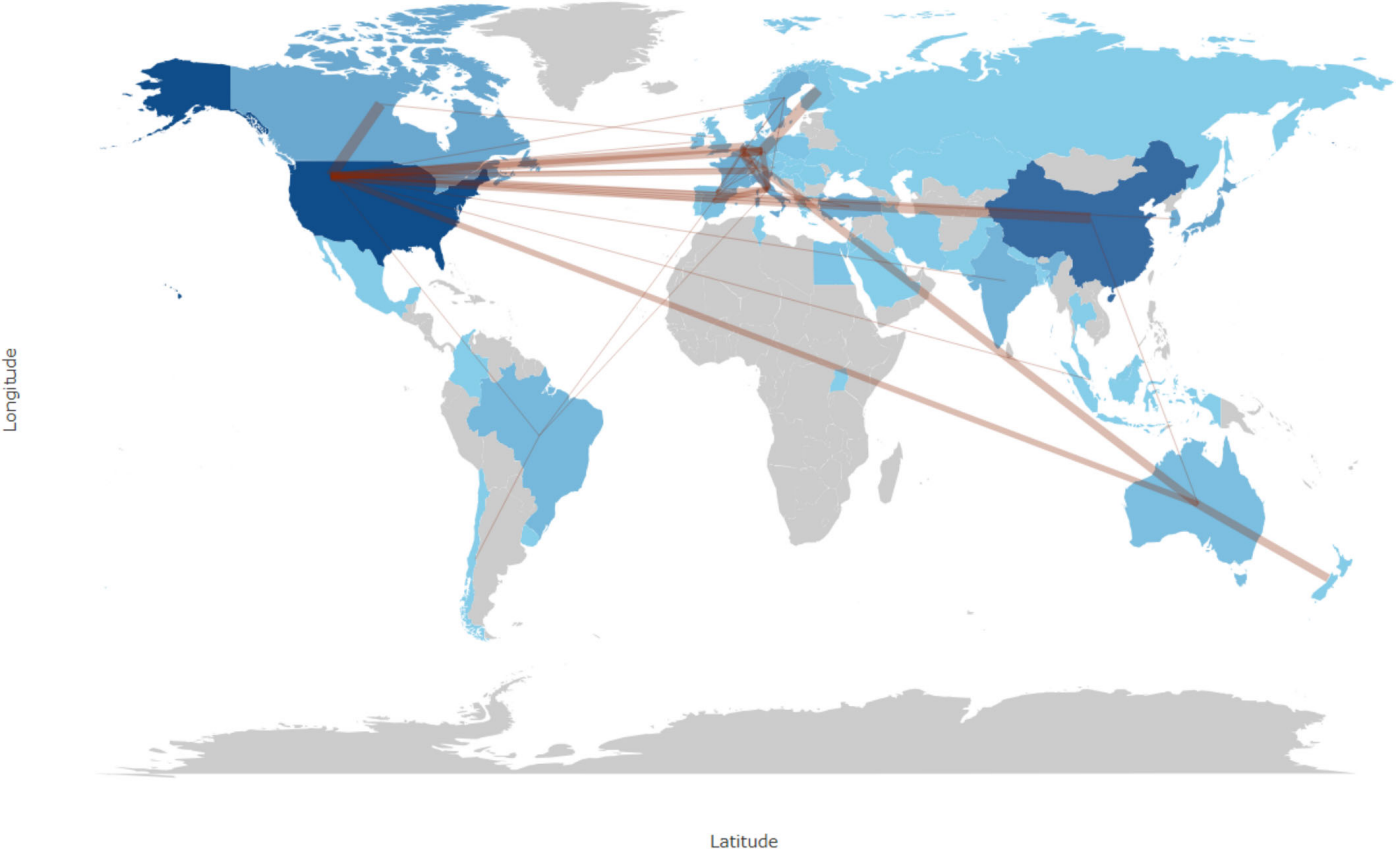
World map of collaboration between countries.

**Figure 6** depicts the international research collaboration network encompassing 38 countries and regions. Within this network, Australia and Belgium are particularly prominent, having established cooperative relationships with over ten different countries each. Furthermore, Brazil, France, and Germany emerge as significant contributors to the network, actively participating in frequent scientific research collaborations with numerous countries.

### 3.9 Analysis of prominent publications

**Figure 7:**
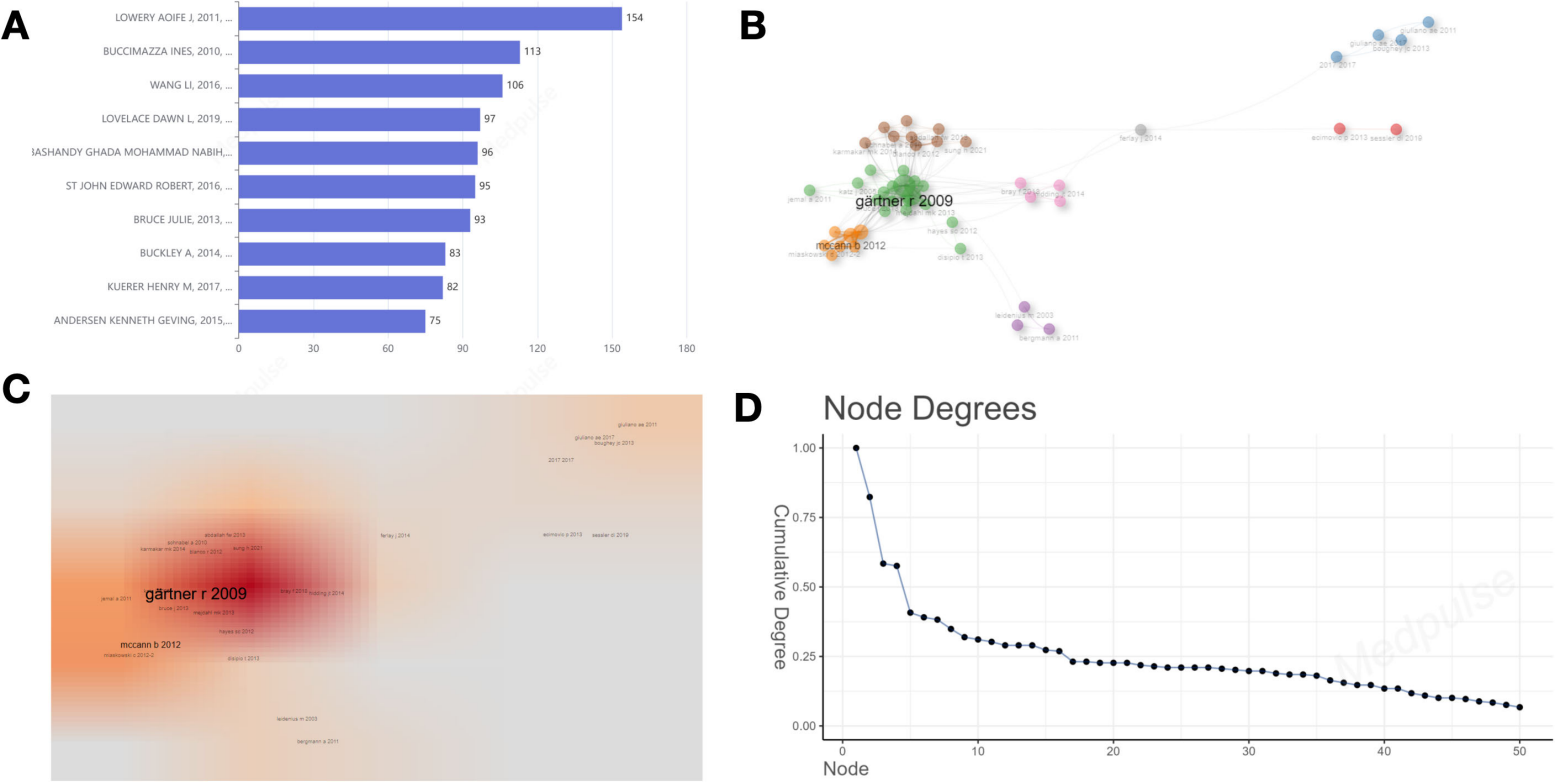
**(A)** The top 10 most globally cited documents. **(B)** Literature co-citation network visualization. **(C)** Literature co-citation density visualization. **(D)** The number of connections per node in the network.

Within the domain of BC surgery, we have highlighted the top ten papers from two distinct perspectives: international citation rankings (**Figure 7A**) (**Table 2**) and impact factor rankings (**Table 3**). The paper “Locoregional Recurrence After Breast Cancer Surgery: A Systematic Review by Receptor Phenotype” is the most cited, with 154 citations. “Perioperative Management May Lead to Less Pain After Breast Cancer Surgery” ranks highest in impact factor, achieving a notable score of 503.1.

**Table 2:** Highly cited literature.

Title	Published Date	Periodical	IF	Quoted
Locoregional recurrence after breast cancer surgery: a systematic review by receptor phenotype.	2011/12/8	BREAST CANCER RESEARCH AND TREATMENT	3	154
Improving breast cancer surgery: a classification and quadrant per quadrant atlas for oncoplastic surgery.	2010/2/9	ANNALS OF SURGICAL ONCOLOGY	3.4	113
Predictors of persistent pain after breast cancer surgery: a systematic review and meta-analysis of observational studies.	2016/7/13	CMAJ: Canadian Medical Association journal = journal de l’Association medicale canadienne	0	106
Long-Term Effects of Breast Cancer Surgery, Treatment, and Survivor Care.	2019/7/20	*Journal of midwifery & women’s health*	0	97
Pectoral nerves I and II blocks in multimodal analgesia for breast cancer surgery: a randomized clinical trial.	2014/11/8	REGIONAL ANESTHESIA AND PAIN MEDICINE	5.1	96
Diagnostic Accuracy of Intraoperative Techniques for Margin Assessment in Breast Cancer Surgery: A Meta-analysis.	2016/7/19	ANNALS OF SURGERY	7.5	95
Psychological, surgical, and sociodemographic predictors of pain outcomes after breast cancer surgery: a population-based cohort study.	2013/10/9	PAIN	5.9	93
Effect of anesthetic technique on the natural killer cell anti-tumor activity of serum from women undergoing breast cancer surgery: a pilot study.	2014/7/11	BRITISH JOURNAL OF ANAESTHESIA	9.1	83
A Clinical Feasibility Trial for Identification of Exceptional Responders in Whom Breast Cancer Surgery Can Be Eliminated Following Neoadjuvant Systemic Therapy.	2017/5/27	ANNALS OF SURGERY	7.5	82
Predictive factors for the development of persistent pain after breast cancer surgery.	2015/7/16	PAIN	5.9	75

**Table 3:** High-scoring literature.

Title	Published Date	Periodical	IF	Quoted
Perioperative management may lead to less pain after breast cancer surgery.	2018/11/27	CA A CANCER JOURNAL FOR CLINICIANS	503.1	2
Nodal Irradiation after Breast-Cancer Surgery in the Era of Effective Adjuvant Therapy.	2015/7/23	NEW ENGLAND JOURNAL OF MEDICINE	96.2	9
Eliminating the breast cancer surgery paradigm after neoadjuvant systemic therapy: current evidence and future challenges.	2020/1/9	ANNALS OF ONCOLOGY	56.7	74
Important considerations prior to elimination of breast cancer surgery after neoadjuvant systemic therapy: Listening to what our patients want.	2020/4/29	ANNALS OF ONCOLOGY	56.7	3
Key role for liquid biopsy in the elimination of breast cancer surgery following neoadjuvant therapy.	2020/12/10	ANNALS OF ONCOLOGY	56.7	0
Patients should be the tipping point of individualizing breast cancer surgery: Commentary on ‘Eliminating the breast cancer surgery paradigm after neoadjuvant systemic therapy: current evidence and future challenges’.	2020/6/1	ANNALS OF ONCOLOGY	56.7	4
Variant mastectomy rates: implications for quality of care in breast cancer surgery.	2010/6/16	JOURNAL OF CLINICAL ONCOLOGY	42.1	1
Reducing treatment decision conflict difficulties in breast cancer surgery: a randomized controlled trial.	2013/7/10	JOURNAL OF CLINICAL ONCOLOGY	42.1	41
Predictors of Unemployment After Breast Cancer Surgery: A Systematic Review and Meta-Analysis of Observational Studies.	2018/5/15	JOURNAL OF CLINICAL ONCOLOGY	42.1	39
Clinical Prediction Model and Tool for Assessing Risk of Persistent Pain After Breast Cancer Surgery.	2017/5/20	JOURNAL OF CLINICAL ONCOLOGY	42.1	38

### 3.10 Co-citation network

**Figures 7B–D** depict the co-citation network within the literature. Co-citation is the simultaneous citation of two references within a single article. In this network, nodes and links represent the citation relationships between referenced works and their co-citation connections. The most significant contribution is attributed to the work of Gärtner (2009), with metrics of Betweenness=294.5934, Closeness=0.0068, and PageRank=0.0654. This is followed by McCann (2012), with Betweenness=93.5772, Closeness=0.0066, and PageRank=0.0333.

### 3.11 Keywords analysis

**Figure 8:**
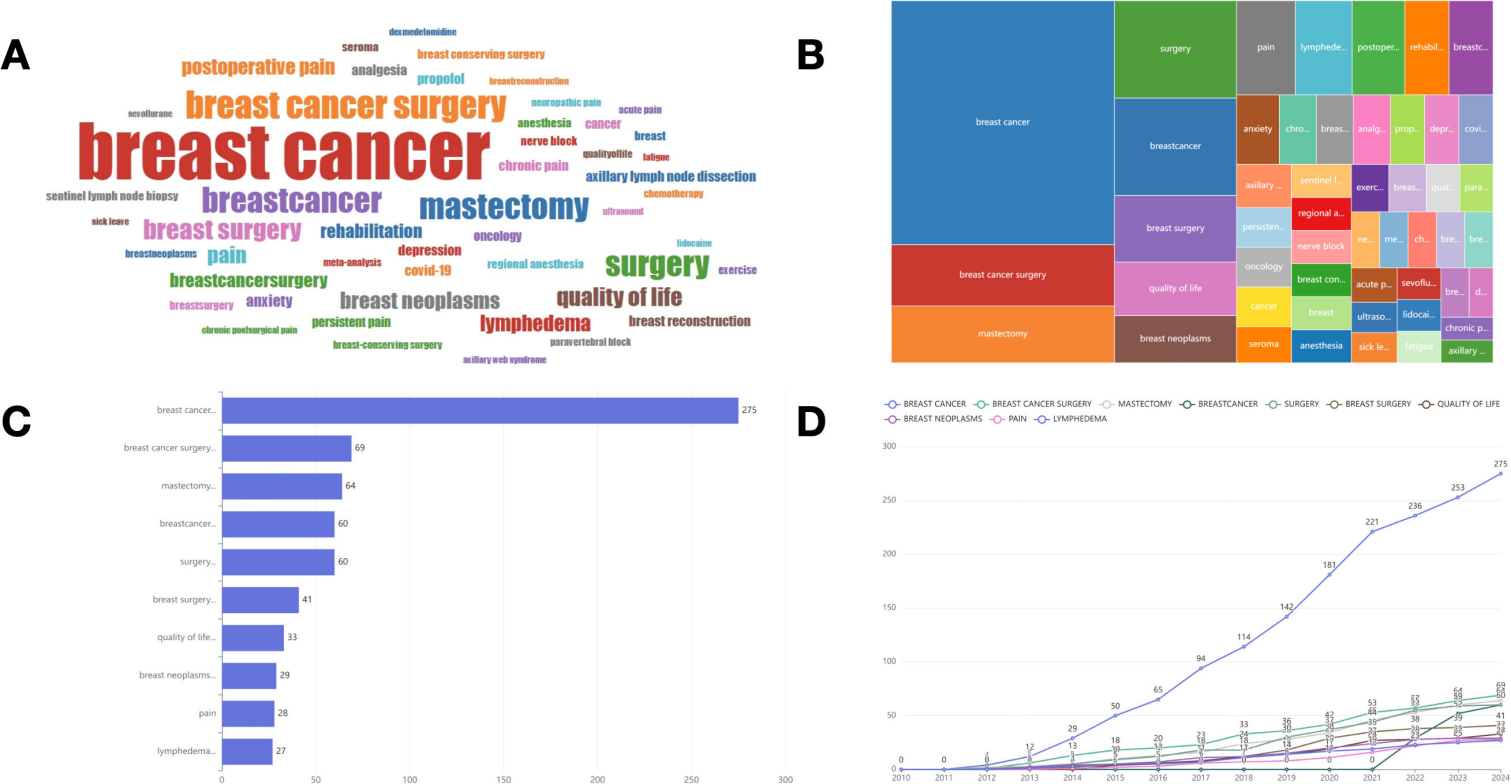
**(A)** World-Cloud for the relevant words. **(B)** Tree map for the relevant words. **(C)** Top 10 most frequent words. **(D)** Change in the frequency of the top 10 keywords over time.

**Figures 8A, B** depict the high-frequency keywords prevalent in the literature of this field, while **Figure 8C** presents the ten most frequently occurring keywords. “Breast cancer” emerges as the most prevalent keyword (frequency = 275), followed by “breast cancer surgery” (frequency = 69), “mastectomy” (frequency = 64), and “surgery” (frequency = 60). These findings align with the results derived from the word cloud analysis. **Figure 8D** illustrates the temporal trends of the top ten keywords based on frequency, highlighting that “breast cancer” consistently remains the most frequent keyword and exhibits the most significant growth trend over time. Since 2022, the term “breastcancer” has gained prominence, demonstrating an increasing trend in annual frequency.

### 3.12 Co-occurrence network

**Figure 9:**
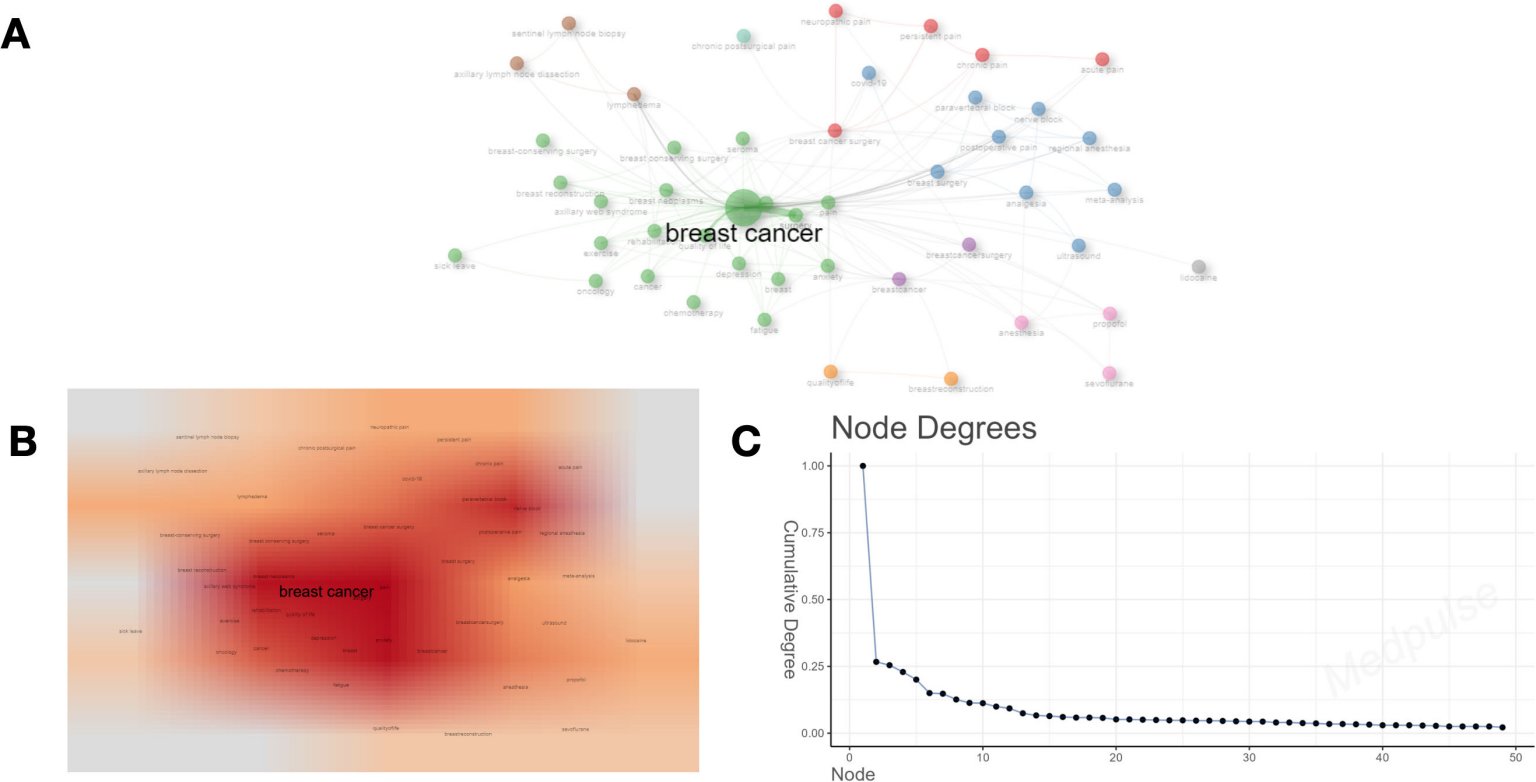
**(A)** Clustering diagram based on keyword analysis. The size of the circle indicates the number of occurrences of the keyword, and the different colors indicate the diversity of the clusters. **(B)** Darker areas indicate higher co-occurrence frequencies or stronger relationship densities. **(C)** The number of connections per node in the network.

We conducted a co-occurrence network analysis of keywords (**Figure 9A**), which illustrates the interconnections among keywords in this domain. The density plot analysis (**Figure 9B**) provides a visualization based on the frequency of keyword occurrences, with “breast cancer” emerging as the most prominent term. Simultaneously, the degree distribution analysis (**Figure 9C**) underscores the particularly significant presence of “breast cancer,” reflecting its central position within the network diagram. **Table 4** presents thematic descriptions and total link strength of keywords, organized into nine clusters: Pain Management Following BC Surgery, Management of Postoperative Pain and Regional Anesthesia (RA), Treatment and Rehabilitation of BC, Surgical Interventions for BC, Quality of Life Among BC Patients in Relation to Breast Reconstruction, Management of Lymphedema and Lymph Node Surgery Following BC Treatment, Anesthesia Management During BC Surgery, Use of Lidocaine in BC Surgery, and Incidence of Chronic Postsurgical Pain Following BC Surgery.

**Table 4:** Thematic co-occurrence clustering.

Cluster	Keywords	Total link strength	Theme description
Cluster 1 (red)	BC surgery	88.8314	Pain Management Following BC Surgery
	chronic pain	2.9049	
	persistent pain	3.5672	
	acute pain	0.0588	
	neuropathic pain	0.0698	
Cluster 2 (blue)	breast surgery	4.1246	Management of Postoperative Pain and RA
	postoperative pain	22.3006	
	analgesia	3.3369	
	covid-19	0.1712	
	nerve block	1.6016	
	regional anesthesia	0.2312	
	meta-analysis	0.486	
	paravertebral block	0.0768	
	ultrasound	0.1682	
Cluster 3 (green)	BC	670.5742	Treatment and Rehabilitation of BC
	mastectomy	109.3482	
	surgery	29.6121	
	quality of life	5.9012	
	breast neoplasms	2.2567	
	pain	32.8954	
	rehabilitation	0.997	
	anxiety	2.3444	
	breast reconstruction	0.0225	
	depression	0.0501	
	cancer	2.2816	
	oncology	0.5896	
	breast	0.0827	
	breast conserving surgery	0.0474	
	seroma	7.3174	
	exercise	0.3335	
	BCS	0.0183	
	chemotherapy	0.0173	
	fatigue	0.1943	
	sick leave	0.0194	
	axillary web syndrome	0.0165	
Cluster 4 (purple)	breastcancer	82.4307	Surgical Interventions for BC
	breastcancersurgery	0.9423	
Cluster 5 (orange)	qualityoflife	0.799	The Quality of Life Among BC Patients In Relation to Breast Reconstruction
	breastreconstruction	0.0152	
Cluster 6 (brown)	lymphedema	8.2616	Management of Lymphedema and Lymph Node Surgery Following BC Treatment
	axillary lymph node dissection	0.0266	
	sentinel lymph node biopsy	0.0242	
Cluster 7 (pink)	propofol	1.1742	Anesthesia management during BC surgery
	anesthesia	2.9493	
	sevoflurane	0.0217	
Cluster 8 (gray)	lidocaine	0.0132	The Use of Lidocaine in BC Surgery
Cluster 9 (cyan)	chronic postsurgical pain	0.0133	The Incidence of Chronic Postsurgical Pain Following BC Surgery

### 3.13 Analysis of trend themes and topic mapping

**Figure 10:**
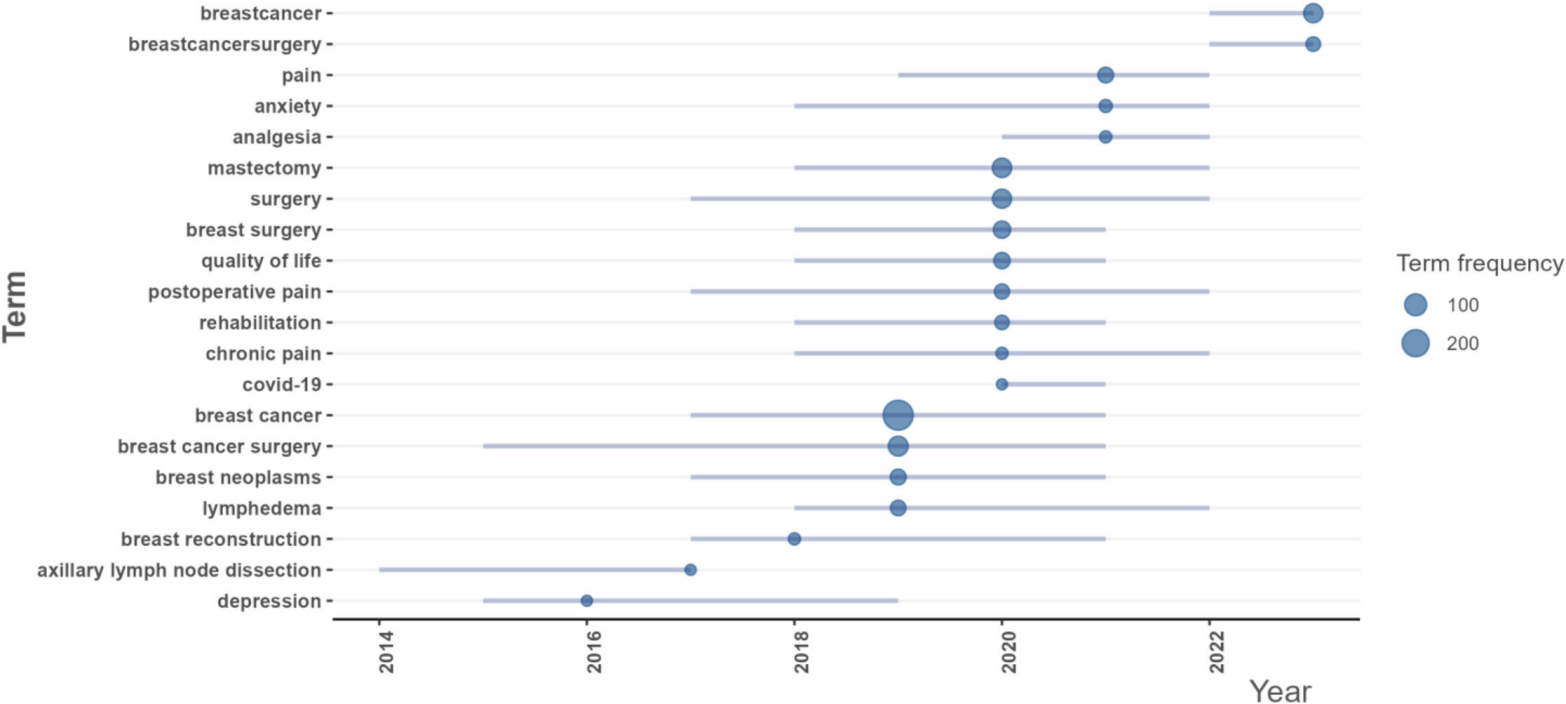
Trend topics authors’ keywords.

**Figure 11:**
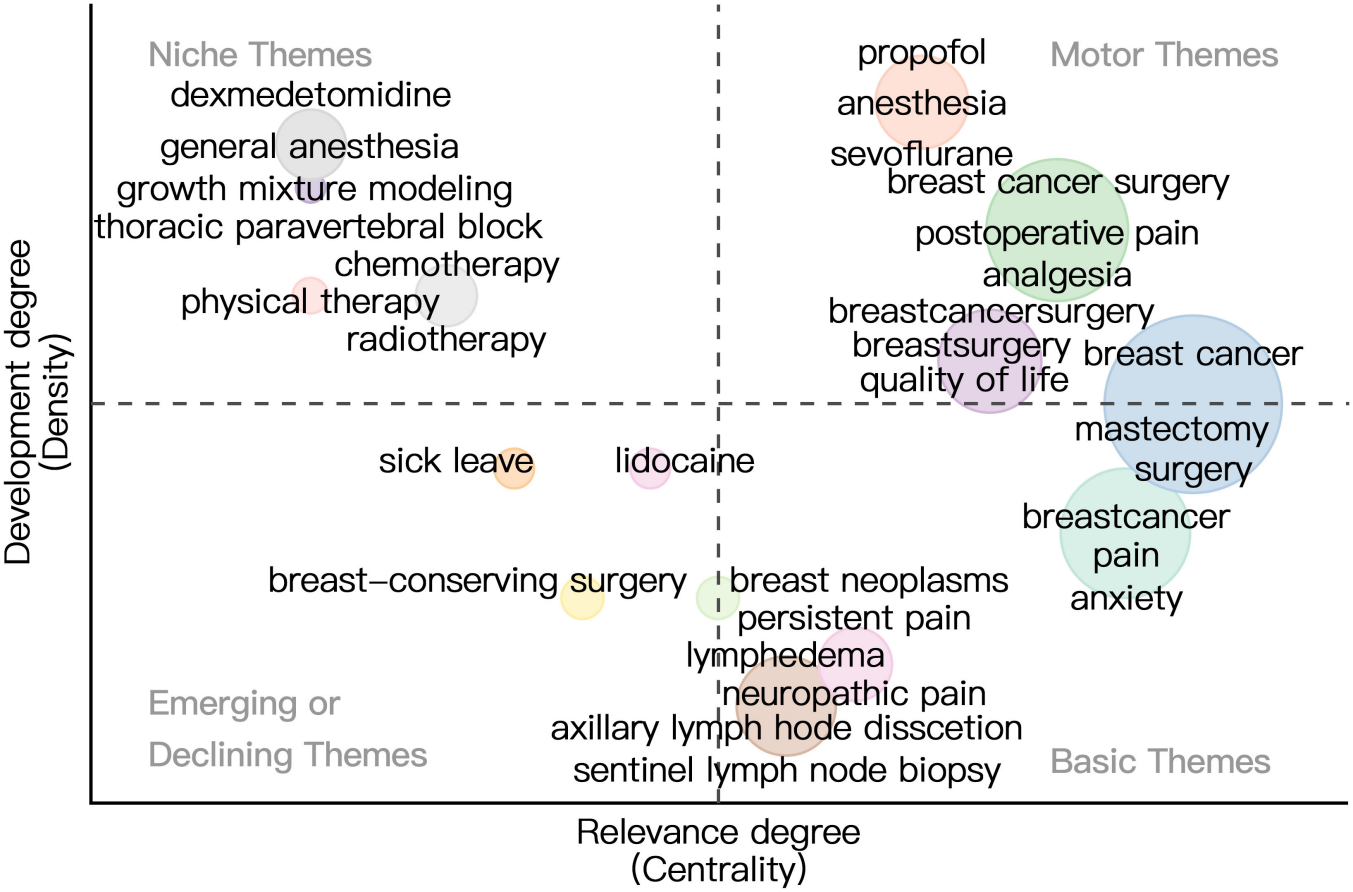
Thematic map. The horizontal axis represents centrality and indicates how relevant the topic is to the field. The vertical axis represents density, which indicates how developed the topic in the field is. Four quadrants are plotted accordingly: Motor Themes, Niche Themes, Emerging or Declining Themes, Basic Themes.

To address the analytical inconsistencies observed across various software tools, we employed the Bibliomtrix software package to more accurately identify research hotspots within the field. **Figure 10** delineates several prominent research themes. In 2019, the primary focal areas included “breast cancer” (frequency=275), “breast cancer surgery” (frequency=69), “breast neoplasms” (frequency=29), and “lymphedema” (frequency=27), corresponding to clusters 1, 3, and 6. In 2020, scholarly investigations predominantly addressed the topics of “mastectomy” (frequency=64), “surgery” (frequency=60), “breast surgery” (frequency=41), “quality of life” (frequency=33), “postoperative pain” (frequency=25), “rehabilitation” (frequency=21), “chronic pain” (frequency=13), and “COVID-19” (frequency=12), aligning with clusters 1, 2, and 3. The emergence of “COVID-19” underscores the critical need to examine the pandemic’s impact on the accessibility and continuity of BC treatment. In 2021, research efforts shifted to focus on “pain” (frequency=28), “anxiety” (frequency=15), and “analgesia” (frequency=13), corresponding to clusters 2 and 3. In 2023, the primary research focal points remained centered on “breastcancer” (frequency = 60) and “breastcancersurgery” (frequency = 21), both of which are associated with cluster 4.By employing co-occurrence network analysis, we can discern the overarching categories of research themes within this domain. Nevertheless, identifying future research trajectories continues to pose significant challenges. We employed the thematic mapping module (**Figure 11**) to analyze current research themes and identify potential future directions in BC surgery. The first quadrant includes key and established themes such as “breast cancer surgery,” “propofol,” “anesthesia,” “sevoflurane,” “postoperative pain,” “analgesia,” and “quality of life.” In contrast, the second quadrant comprises themes such as “dexmedetomidine,” “general anesthesia,” “growth mixture modeling,” “thoracic paravertebral block,” “chemotherapy,” “physical therapy,” and “radiotherapy,” which, although gaining popularity, are of comparatively lesser relevance to the field. The third quadrant encompasses themes such as “sick leave,” “lidocaine,” and “breast-conserving surgery,” which are characterized as underdeveloped, emerging, or declining. The fourth quadrant includes themes like ‘breast cancer’ ‘pain’ ‘anxiety’ ‘persistent pain’ ‘lymphedema’ ‘neuropathic pain’ ‘axillary lymph node dissection’ and ‘sentinel lymph node biopsy. ‘These themes are considered fundamental and significant to the field, yet they remain in a nascent stage of development. The terms “mastectomy” and “surgery” appear in both the first and fourth quadrants, indicating their significance as central development hotspots. Notably, all keywords located in the fourth quadrant are associated with clusters 1, 3, 4, and 6.Based on this integration of findings, it can be inferred that “breast cancer,” “pain,” “anxiety,” “lymphedema,” “mastectomy,” and “surgery” potentially represent the research frontier in this field.

## 4 Discussion

### 4.1 Research status

This study employed software tools including R language, CiteSpace, and VOSviewer to perform a comprehensive visual analysis of 1,195 scholarly articles related to BC surgery, sourced from the PubMed database spanning the years 2010 to 2024. The analysis encompassed various dimensions such as publication and citation metrics, collaboration network analysis, journal evaluation, author and institutional profiling, country-specific contributions, identification of seminal literature, keyword examination, and the exploration of research hotspots within the field.

The total number of publications in this field is increasing, but citation frequency is declining. This pattern implies an increasing research interest in the area, which may also reflect a potential decline in the quality of papers or a saturation of the research domain, thereby hindering the ability of new studies to achieve significant breakthroughs. The observed decline in both publications and citations in 2024 could be attributed to the temporal limitations of the dataset, as the data collection was concluded on August 7th. China, the United States, and Japan are the leading publishing countries, with the United States showing the most frequent international collaborations. Conversely, Spain requires enhancement in its international cooperative efforts. Furthermore, an analysis of the national cooperation network indicates that Australia and Belgium maintain the most robust cooperative relationships. Christine Miaskowski, Steven M. Paul, and Jon D. Levine are among the authors with the highest publication output, with significant contributions in 2012 and 2014. This prominence may be attributed to advancements in research methodologies or techniques that enhanced research efficiency. The journals *Annals of Surgical Oncology*, *Breast Cancer Research and Treatment*, and the *British Journal of Surgery* have the highest publication counts, with each showing a trend of rising annual publication rates. The article “Locoregional Recurrence After Breast Cancer Surgery: A Systematic Review by Receptor Phenotype” (17) has been cited 154 times, whereas “Perioperative Management May Lead to Less Pain After Breast Cancer Surgery” (18) has an impact factor of 503.1.Both studies provide fresh insights into BC surgery and propose directions for future research. The placement of the two documents, Gärtner R (2009) (19) and McCann B (2012) (20), within the co-citation network suggests that they possess substantial influence and centrality within the scholarly discourse on BC surgery. These works have significantly contributed to the advancement of the field. The aforementioned studies demonstrate a consistent trajectory of progress in BC surgery research.

### 4.2 Research hotspots and trends

Keywords serve as concise representations of the central themes within the literature. An analysis of these keywords revealed that “breast cancer surgery,” “mastectomy,” and “surgery” were among the most frequently occurring terms. By integrating this analysis with studies on theme co-occurrence, thematic trends, and thematic mapping, we have identified “breast cancer,” “pain,” “anxiety,” “lymphedema,” “mastectomy,” and “surgery” as the principal research domains in this field. In the following sections, we will explore the research hotspots and trends associated with each of these five thematic clusters.

### 4.3 Cluster one: pain management following BC surgery

Effective pain management following BC surgery constitutes a critical component of patient care, given the prevalence of pain experienced by patients postoperatively. Post-surgical pain can be classified into acute pain, which generally arises immediately after the procedure, and chronic pain, which may develop progressively over time. Chronic pain post-BC surgery is characterized by its persistence and affects approximately 30% of patients, frequently exhibiting neuropathic features (21). The progression from acute to chronic pain represents a critical concern, given its potential to result in prolonged suffering and a diminished quality of life. Research suggests that between 10% and 50% of patients may experience chronic post-surgical pain, with contributory factors including nerve damage and inflammation being pivotal in its development (22). A thorough grasp of the mechanisms driving this transition is crucial for developing effective pain management strategies.

Effective pain management includes both pharmacological and non-pharmacological approaches. Opioids are commonly used for acute pain management, but extended use can lead to negative effects such as opioid-induced hyperalgesia (23). As a result, there is a growing emphasis on opioid-sparing pain management strategies, including the use of non-pharmacological methods like mindfulness-based stress reduction and acupuncture (22).

Furthermore, the identification of predictors for persistent postoperative pain is of paramount importance. Existing research indicates that factors such as chronic preoperative pain, psychological resilience, and the surgical techniques employed can substantially affect pain outcomes following surgery (24, 25). For example, individuals experiencing chronic pain prior to surgery are at an increased risk of moderate to severe postoperative pain, underscoring the necessity for comprehensive preoperative assessments and the implementation of targeted intervention strategies (25).

In conclusion, effective pain management following BC surgery necessitates a holistic approach that addresses both acute and chronic pain. By comprehensively understanding the associated risk factors and underlying mechanisms, healthcare providers can develop and implement individualized strategies aimed at enhancing patient treatment outcomes and mitigating the prevalence of persistent pain.

### 4.4 Cluster two: management of postoperative pain and RA

Effective postoperative pain management is a critical component of patient care, especially in the context of breast surgery, where moderate to severe pain can significantly impact patient recovery and overall satisfaction. Pectoral nerve blocks and erector spinae plane blocks are effective RA techniques for analgesia in breast surgery patients. Systematic reviews and network meta-analyses have indicated that these RA techniques are superior to traditional local anesthetic infiltration in managing early postoperative resting pain (26).

Moreover, the integration of RA into a multimodal analgesia regimen can substantially decrease reliance on opioids, a critical consideration given the increased awareness of opioid-related complications and the current opioid crisis. RA techniques not only alleviate pain but also facilitate faster recovery and shorter discharge times, aligning with Enhanced Recovery After Surgery protocols. These protocols underscore the significance of minimizing perioperative stress and optimizing pain management to enhance patient outcomes (27).

Amidst the ongoing challenges of the COVID-19 pandemic, integrating RA into postoperative pain management is essential for healthcare systems. This method addresses the immediate requirements of patients undergoing breast surgery while simultaneously enhancing surgical care pathways and optimizing overall patient outcomes within a swiftly evolving healthcare landscape (28). A systematic review indicates that paravertebral blocks significantly reduce both postoperative analgesic requirements and the incidence of postoperative nausea and vomiting compared to general anesthesia (29).

Furthermore, the implementation of ultrasound-guided paravertebral blocks has been shown to enhance the success rate of the procedure, resulting in improved analgesic outcomes and diminished opioid consumption (30). In a randomized controlled trial, ultrasound-guided paravertebral blocks demonstrated superior anesthetic and perioperative analgesic efficacy compared to traditional anatomical landmark techniques (31). This technological advancement enhances patient comfort and facilitates expedited recovery and discharge (32). Research suggests that managing postoperative pain with paravertebral blocks can reduce the occurrence of persistent post-surgical pain syndrome (33). The results underscore the significance of integrating RA techniques, particularly ultrasound-guided paravertebral blocks, into multimodal analgesia regimens to optimize postoperative pain management and enhance the overall recovery experience for patients undergoing diverse surgical procedures (34).

In general, the incorporation of RA and multimodal analgesia into postoperative pain management protocols can enhance patient comfort, contribute to improved surgical outcomes, and mitigate opioid-related complications.

### 4.5 Cluster three: treatment and rehabilitation of BC

The management and rehabilitation of BC involve a comprehensive array of surgical and non-surgical interventions designed to enhance treatment outcomes and patient quality of life. Mastectomy, a principal surgical intervention, entails the excision of one or both breasts, primarily to eradicate malignant tissue. The decision between undergoing a mastectomy and opting for BCS can profoundly influence the patient’s quality of life following treatment. Research indicates that although both surgical interventions can produce satisfactory outcomes, patients undergoing BCS frequently report superior quality of life metrics, especially concerning body image and emotional well-being (35, 40).

Conversely, post-mastectomy patients may encounter several complications, such as seroma formation, characterized by fluid accumulation at the surgical site. This condition can lead to discomfort and may necessitate further medical intervention, thereby impacting the overall recovery process and quality of life (38). Comprehending the implications of surgical complications, such as seroma, is essential for both patients and healthcare providers in the formulation of treatment plans. Research indicates that although mastectomy may be linked to specific complications, the overall effect on quality of life is contingent upon individual factors, including the type of reconstruction undertaken and the patient’s psychological resilience (36, 41).

Furthermore, the psychosocial dimensions of BC treatment warrant significant consideration. The decision to pursue mastectomy or breast reconstruction is frequently shaped by variables including patient age, individual preferences, and the risk-benefit profile of each surgical intervention. Notably, in older patients, the option of breast reconstruction post-mastectomy can substantially enhance quality of life, notwithstanding initial reluctance from both patients and surgeons (36, 37).

In conclusion, the interplay between BC treatment, surgical procedures like mastectomy, and subsequent rehabilitation is complex. It is imperative for healthcare professionals to thoroughly discuss with patients the available treatment options, potential complications such as seroma, and the anticipated effects on quality of life. This comprehensive approach facilitates informed decision-making by patients, ensuring alignment with their personal values and health objectives (39, 42).

### 4.6 Cluster four: surgical interventions for BC

Surgical management of BC primarily focuses on techniques such as BCS and mastectomy. BCS commonly known as lumpectomy, is the standard procedure for early-stage BC, allowing tumor removal while preserving as much breast tissue as possible (43). This approach significantly improves cosmetic outcomes and patient quality of life (45). Accurate localization of tumors during BCS continues to present significant challenges, particularly in cases involving small or non-palpable lesions. This difficulty can result in incomplete tumor excision and elevated rates of re-excision (43).

Recent advancements in surgical methodologies and technologies have been directed toward improving the precision of BC surgeries. Notably, the integration of fiber-optic photoacoustic guidance with augmented reality has demonstrated potential in enhancing tumor localization accuracy during surgical procedures, thereby potentially decreasing the necessity for re-excision (43). Advanced imaging techniques, such as quantitative micro-elastography and Cerenkov luminescence imaging, are being explored for their ability to identify residual cancer in the surgical cavity in real-time, potentially improving surgical results (47, 49).

In parallel, the management of axillary lymph nodes is undergoing significant transformation, with a discernible trend toward minimally invasive surgical approaches. The transition from routine axillary clearance to SLNB marks a pivotal shift, contributing to reduced morbidity and improved patient quality of life (45). This is particularly significant as a growing number of early-stage BC patients benefit from personalized treatment strategies that reduce invasiveness while ensuring effective cancer management (45).

The approach to treating ductal carcinoma *in situ* (DCIS) is undergoing changes. Studies indicate that many patients undergoing BCS for DCIS may face a second breast event, necessitating further surgical procedures (44). Comprehending the patterns of care and treatment modalities for SBE is essential for optimizing patient outcomes and informing future clinical practices (44).

The surgical management of BC is undergoing constant evolution, driven by ongoing research and technological advancements that seek to enhance precision, minimize complications, and improve the overall patient experience (46, 48, 50).

### 4.7 Cluster six: management of lymphedema and lymph node surgery following BC treatment

The management of lymphedema and the surgical treatment of lymph nodes are essential aspects of patient care following BC therapy. Surgical procedures for axillary lymph nodes include axillary lymph node dissection (ALND) and Sentinel lymph node biopsy (SLNB). ALND has traditionally been the standard surgical approach for patients with node-positive BC. However, recent research indicates that SLNB may provide a less invasive option with a reduced risk of complications, including lymphedema (51).

Lymphedema is a common complication of axillary surgery that can significantly impact patients’ quality of life. Research indicates that SLNB is associated with a lower incidence of lymphedema than ALND (52). This finding is especially pertinent for women with node-negative BC, as SLNB provides an effective method for axillary staging while concurrently minimizing the risk of developing lymphedema (53).

The management of postoperative lymphedema generally encompasses a multifaceted approach, including physical therapy, the use of compression garments, and instruction in patient self-care methodologies. Furthermore, current clinical trials are investigating the potential of axillary radiation therapy as an alternative to surgical clearance in specific patient cohorts, which may contribute to a decreased incidence of lymphedema (54).

A thorough grasp of lymph node surgery and lymphedema treatment impacts is crucial for enhancing therapeutic results and quality of life in BC survivors. As the field progresses, it is imperative to develop individualized treatment plans that take into account the specific circumstances and preferences of patients in order to effectively address the complexities associated with BC care (55, 56).

### 4.8 Limitations

This study is constrained by its reliance on the PubMed database, which may introduce potential publication bias. Furthermore, the cutoff date for the statistics is August 7, 2024, potentially leading to incomplete publication data for the year 2024 and consequently impacting the reliability of the reported findings. Moreover, the analysis may exhibit a bias toward mastectomy or lumpectomy, potentially neglecting other critical dimensions of BC surgery, such as reconstructive procedures and the psychological ramifications of surgical choices on patients. This narrow focus may limit the applicability of the study’s findings to the wider context of BC treatment.

## 5 Conclusion

This study utilized bibliometric tools such as R language, CiteSpace, and VOSviewer for a visual analysis of literature on BC surgery. This approach provided a comprehensive overview of the research landscape, highlighting key themes and emerging trends in the field over the past 25 years. The analysis revealed that China, the United States, the University of California, author Christine Miaskowski, the journal *Annals of Surgical Oncology*, and two seminal papers have significantly influenced the field’s development. We identified key thematic areas, namely “BC,” “pain,” “anxiety,” “lymphedema,” “mastectomy,” and “surgery,” and analyzed the five clusters associated with these themes, offering novel insights for research in this domain. This study is timely in guiding future research trajectories in the field, although it is not without limitations. Future investigations should broaden the range of databases consulted, incorporate a greater number of high-quality articles, and enhance the data support for research pertaining to BC surgery.

## Data Availability

The original contributions presented in the study are included in the article/supplementary material. Further inquiries can be directed to the corresponding author.
