# Corrigendum: Research progress of breast cancer surgery during 2010–2024: a bibliometric analysis

**DOI:** 10.3389/fonc.2025.1550434

**Published:** 2025-01-31

**Authors:** Jiawei Kang, Nan Jiang, Munire Shataer, Tayier Tuersong

**Affiliations:** ^1^ Department of Clinical Medicine, Xinjiang Medical University, Ürümqi, Xinjiang, China; ^2^ Department of Histology and Embryology, Basic Medical College of Xinjiang Medical University, Ürümqi, Xinjiang, China; ^3^ Department of Pharmacy, Xinjiang Key Laboratory of Neurological Diseases, Xinjiang Clinical Research Center for Nervous System Diseases, Second Affiliated Hospital of Xinjiang Medical University, Ürümqi, Xinjiang, China

**Keywords:** breast cancer surgery, bibliometric analysis, research hotspots, international collaboration, publication patterns

In the published article, the reference for 14 was incorrectly written as “van Eck NJ, Waltman L. Software survey: VOSviewer, a computer program for bibliometric mapping. Scientometrics. (2010) 84:523–38. doi: 10.1007/s11192-009-0146-3”. It should be “Ogunsakin RE, Ebenezer O, Ginindza TG. A bibliometric analysis of the literature on Norovirus disease from 1991-2021. *Int J Environ Res Public Health*. (2022) 19(5):2508. doi: 10.3390/ijerph19052508”.

In the published article, the reference for 15 was incorrectly written as “Waltman L, van Eck NJ. A new methodology for constructing a publication-level classification system of science. J Assoc Inf Sci Technol. (2012) 63:2378–92. doi: 10.1002/asi.22693”. It should be “Musa HH, Musa TH. A systematic and thematic analysis of the top 100 cited articles on mRNA vaccine indexed in Scopus database. *Hum Vaccin Immunother*. (2022) 18(6):2135927. doi: 10.1080/21645515.2022.2135927”.

In the published article, the reference for 16 was incorrectly written as “Chen C. Science mapping: A systematic review of the literature. J Data Inf Sci.(2017) 2:1–40. doi: 10.1515/jdis-2017-0006]”. It should be “Volpe S, Mastroleo F, Krengli M, Jereczek-Fossa BA. Quo vadis radiomics? Bibliometric analysis of 10-year Radiomics journey. *Eur Radiol*. (2023) 33(10):6736–45. doi: 10.1007/s00330-023-09645-6”.

In the published article, there was an error. The method description was inaccurate.

A correction has been made to **Abstract**, *Methods*, Paragraph 1. This sentence previously stated:

“Employing the “bibliometrix” package in the R programming language,

alongside VOSviewer and CiteSpace software”

The corrected sentence appears below:

“Employing the “bibliometrix” package in the R software”

A correction has been made to **Abstract**, *Methods*, Paragraph 1. This sentence previously stated:

“The analysis encompassed publication trends, collaborative networks, journal evaluation, author and institutional assessments, country-specific analyses, keyword exploration, and the identification of research hotspots.”

The corrected sentence appears below:

“The analysis encompassed publication trends, collaborative networks, co-citation networks, co-occurrence networks, journal evaluation, prominent publications, author and institutional assessments, country-specific analyses, keyword exploration, and the identification of research hotspots.”

In the published article, there was an error.

A correction has been made to **Methods**, *2.2 Data analysis*, Paragraph 1. This sentence previously stated:

“We conducted a bibliometric analysis of the collected data using the”bibliometrix” package in R (version 4.3.1, http://www.bibliometrix.org) (13). This package enabled the extraction of critical information and the construction of co-occurrence networks encompassing countries, institutions, journals, and authors. Furthermore, it facilitated thematic evolution analysis and the development of a global publication distribution network. In addition, VOSviewer software was employed for the visualization of collaboration and coword networks (14, 15). CiteSpace (version 5.8.R2) was employed to analyze keyword co-occurrence and citation networks, revealing research trends and knowledge structures (16).”

The corrected sentence appears below:

“The content should be changed to the following: This study predominantly utilizes the R package “bibliometrix” (version 4.2.3) (13) (accessible at https://www.bibliometrix.org) for conducting bibliometric analysis. Data analysis is performed using R code in conjunction with the Bibliometrix package (R version 4.2.0) (14)(15). The initial data interpretation is facilitated through the “biblioAnalysis()” command and the “summary()” function within the Bibliometrix package (16). Collaboration networks are examined using the “metaTagExtraction” and “Biblionetwork” commands, with subsequent graphical representation achieved via the “Networkplot” command. Furthermore, the “Biblioshiny()” command is employed for analyses pertaining to national scientific collaboration, institutional collaboration networks, keyword analysis, co-occurrence network synthesis, and thematic map analysis.”

A correction has been made to **Results**, *3.13 Analysis of trend themes and topic mapping*, Paragraph 1. This sentence previously stated:

“To address the analytical inconsistencies observed across various software tools, we employed the Bibliomtrix software package to more accurately identify research hotspots within the field”

The corrected sentence appears below:

“We employed the “bibliomtrix” software package to more accurately identify research hotspots within the field”

A correction has been made to **Discussion**, *4.1 Research status*, Paragraph 1. This sentence previously stated:

“This study employed software tools including R language, CiteSpace, and VOSviewer to perform a comprehensive visual analysis of 1,195 scholarly articles related to BC surgery”

The corrected sentence appears below:

“This study employed “bibliometrix” package to perform a comprehensive visual analysis of 1,195 scholarly articles related to BC surgery”

A correction has been made to **Discussion**, *4.8 Limitations*, Paragraph 1. This sentence previously stated:

“the analysis may exhibit a bias toward mastectomy or lumpectomy, potentially neglecting other critical dimensions of BC surgery, such as reconstructive procedures and the psychological ramifications of surgical choices on patients. This narrow focus may limit the applicability of the study’s findings to the wider context of BC treatment.”

The corrected sentence appears below:

“this study sought to incorporate literature of high relevance to BC surgery to enhance the credibility of the research findings. However, this focus may have resulted in the exclusion of other pertinent literature related to BC surgery. We recognize this limitation and commit to examining a more comprehensive array of literature in future research endeavors.”

A correction has been made to **Conclusion**, Paragraph 1. This sentence previously stated:

“This study utilized bibliometric tools such as R language, CiteSpace, and VOSviewer for a visual analysis of literature on BC surgery.”

The corrected sentence appears below:

“This study utilized “bibliometrix” package for a visual analysis of literature on BC surgery.”

The authors apologize for these errors and state that this does not change the scientific conclusions of the article in any way. The original article has been updated.

